# Pattern recognition in menstrual bleeding diaries by statistical cluster analysis

**DOI:** 10.1186/1472-6874-9-21

**Published:** 2009-07-16

**Authors:** Christoph Gerlinger, Jens Wessel, Gerd Kallischnigg, Jan Endrikat

**Affiliations:** 1 Global Clinical Statistics, Bayer Schering Pharma AG, Müllerstraße 178, 13342 Berlin, Germany; 2 Global Medical Affairs Women's Healthcare, Bayer Schering Pharma AG, Berlin, Germany; 3 Klinik für Geburtsmedizin, Charité, Campus Virchow-Klinikum, Humboldt Universität, Berlin, Germany; 4 Institute of Health Sciences, Berlin University of Technology, Berlin, Germany; 5 Bayer Inc., Toronto, Ontario, Canada; 6 Klinik für Frauenheilkunde, Geburtshilfe und Reproduktionsmedizin, Universität des Saarlands, Homburg (Saar), Germany

## Abstract

**Background:**

The aim of this paper is to empirically identify a treatment-independent statistical method to describe clinically relevant bleeding patterns by using bleeding diaries of clinical studies on various sex hormone containing drugs.

**Methods:**

We used the four cluster analysis methods single, average and complete linkage as well as the method of Ward for the pattern recognition in menstrual bleeding diaries.

The optimal number of clusters was determined using the semi-partial R^2^, the cubic cluster criterion, the pseudo-F- and the pseudo-t^2^-statistic. Finally, the interpretability of the results from a gynecological point of view was assessed.

**Results:**

The method of Ward yielded distinct clusters of the bleeding diaries. The other methods successively chained the observations into one cluster. The optimal number of distinctive bleeding patterns was six. We found two desirable and four undesirable bleeding patterns. Cyclic and non cyclic bleeding patterns were well separated.

**Conclusion:**

Using this cluster analysis with the method of Ward medications and devices having an impact on bleeding can be easily compared and categorized.

## Background

Hormonal contraceptives and other treatments with impact on the endometrium alter a woman's natural menstrual bleeding pattern [1]. Any change in the bleeding pattern has a major impact on the individual's quality of life. An unsatisfactory bleeding pattern is one of the major causes for stopping treatment with sex hormones, e.g. for contraception, the treatment of menopausal symptoms, or endometriosis.

An analysis of bleeding patterns is required by drug regulatory agencies such as the EMEA and the FDA in addition to an analysis of efficacy and safety. Although the regulatory requirements for safety and efficacy of hormonal preparations such as contraceptives or hormone replacement therapies are well defined, e.g. [2], the EMEA's guideline on contraceptives [3] requires only that the bleeding pattern is studied in an active controlled study but does not specify how. The EMEA's guideline on hormone replacement therapy [4] is not any more specific.

The aim of this paper is to empirically identify a treatment-independent statistical method to describe clinically relevant bleeding patterns by using bleeding diaries of clinical studies on various sex hormone containing drugs.

## Methods

We analyzed bleeding dairies that were kept in clinical trials involving various products used for hormonal fertility control, hormone replacement therapy and endometriosis. Mono-preparations as well as combined preparations were included. Estrogens, e.g., estradiol, estradiolvalerate or ethinylestradiol and a large variety of modern progestins, e.g, levonorgestrel, desogestrel, dienogest or drospirenone, were the hormonal components of the drugs.

All trials were performed according to the principles of the Declaration of Helsinki [5], the laws applicable in the respective countries, and "Good Clinical Practices" (GCP) [6]. All clinical studies have been approved by the competent ethics committees. The clinical trials were sponsored by Bayer Schering Pharma AG or one of its subsidiaries.

**Figure 1:**
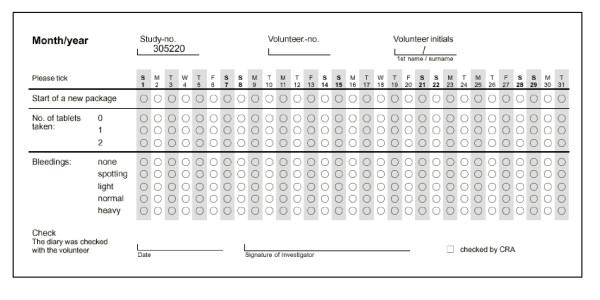
**Example of bleeding diary**. From Bayer Schering Pharma AG's study 305220

The definitions of bleeding intensities that were recorded daily in the bleeding diaries (see Figure 1) were slightly different in the various studies. For the purpose of this analysis, the bleeding intensity categories have been standardized according to WHO terminology [7] as "none", "spotting", and "bleeding". "Spotting" is defined as any vaginal bleeding that does not require the use of sanitary protection such as tampons or pads. "Bleeding" is defined as vaginal bleeding that requires the use of sanitary protection. "None" is defined as neither "Spotting" nor "Bleeding" on that day. These definitions are independent of whether sanitary protection was actually used or not. For the purpose of the cluster analyses, the bleeding intensity scores 0 for "none", 1 for "spotting", and 2 for "bleeding" were used.

All cluster analysis algorithms implemented in SAS^® ^Software [8] require complete data. Therefore, we imputed single missing entries in the bleeding diaries by the maximum of the bleeding intensities of the preceding and the following day. We included all diaries that had a length of at least 90 days in our analyses. This length was chosen to comply with the definition of the reference period length of the WHO [7]. In summary, the dataset consisted of one record per woman with ninety score variables giving the bleeding intensity score for each day.

The bleeding diary data was analyzed using different agglomerative hierarchical cluster analyses because these methods do not require previous knowledge as for example a discriminant analysis. The bleeding patterns in the diaries should be found by unsupervised pattern recognition [9]. As there is no single optimal cluster analysis procedure, we analyzed the data using the single linkage method [10,11], the complete linkage method [12], the average linkage method [13], and the method of Ward [14].

As the number of different bleeding patterns was unknown a priori, we used the semi-partial R^2 ^[8], the cubic cluster criterion [15], the pseudo-F- and the pseudo-t^2^-statistic [8] to derive the optimal number of clusters. Finally, we assessed whether the results of the cluster analyses could be interpreted from a gynecological perspective.

## Results

The clinical databases contained bleeding diaries of 5602 women. Of these 3246 (57.9%) women were treated with hormone replacement therapy after menopause, 2035 (36.3%) were aged 18 to 35 and took an oral contraceptive, and 321 (5.7%) were treated for endometriosis. A total of 4612 (82.3%) diaries were included in our analyses because they covered at least 90 consecutive days. An exploratory data analysis of the bleeding diaries revealed that 1288 (27.9%) of the 4612 women in our dataset never bled during the 90 days analyzed. On the other hand, 3172 (68.8%) women had a unique bleeding pattern that occurred only once in the dataset.

**Figure 2:**
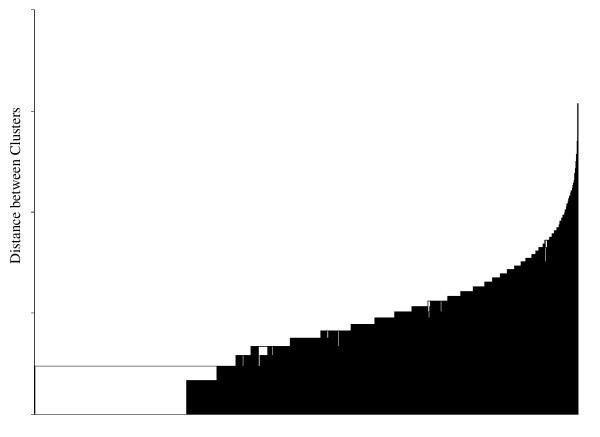
**Dendrogramm of the single linkage method**.

**Figure 3:**
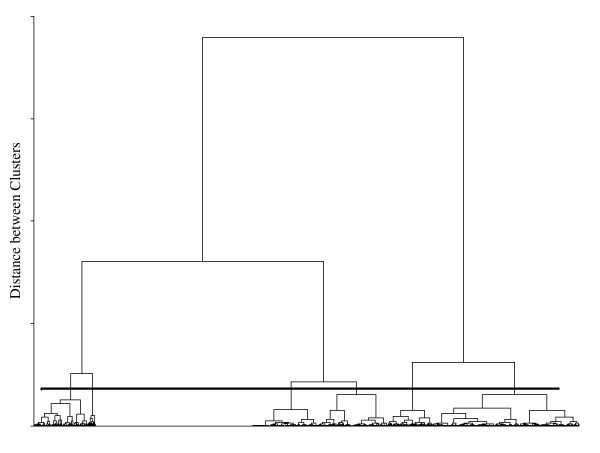
**Dendrogramm of the method of Ward**. Note: The six clusters determined by the horizontal line are -from left to right- the clusters number 6, 5, 3, 4, 2, and 1.

The cluster analyses using the single linkage, complete linkage, and average linkage methods did not produce clinically interpretable results. With all three methods the effect of chaining occurred where the observations are successively joined into a single large cluster (see Figure 2). On the other hand, the method of Ward yielded a clear separation of the bleeding diaries (see Figure 3) into distinct clusters. According to the semi-partial R^2^, the cubic cluster criterion, the pseudo-F, and the pseudo-t statistic, the solutions with three, four, six, and twelve clusters could be of clinical relevance.

Comparing these solutions, we found out, that the solution with six clusters was the best to be interpreted clinically. Three and four cluster did not distinguish the different bleeding patterns to enough detail whereas twelve clusters provided no more clinically useful insight than six clusters. This solution is depicted in Figure 3 by the solid horizontal line.

**Figure 4:**
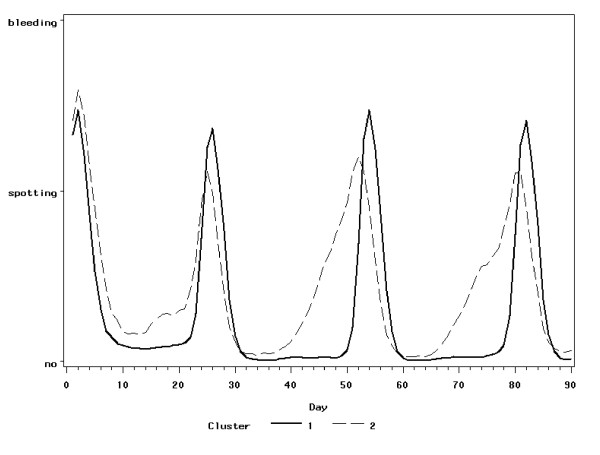
**Cyclic bleeding patterns – mean bleeding intensity**.

**Figure 5:**
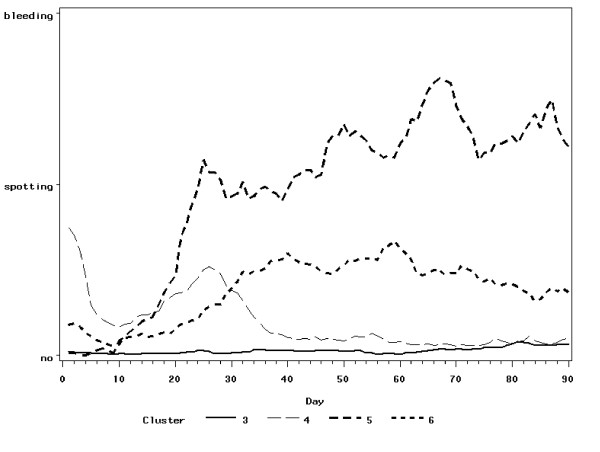
**Continuous bleeding patterns – mean bleeding intensity**.

The cluster analysis revealed two clusters of cyclic bleeding patterns containing 1235 and 386 diaries (see Figure 4) and four clusters of non-cyclic bleeding patterns containing 1880, 590, 71, and 450 diaries (see Figure 5).

The cyclic bleeding patterns are clearly separated into a desirable bleeding pattern (number 1 in Figure), which is characterized by the regular monthly bleeding and a very low frequency of intracyclic bleeding during hormone intake, and an undesirable bleeding pattern (number 2 in Figure 4), which is characterized by a less regular monthly bleeding and a higher frequency of intracyclic bleeding, starting always in the middle of the cycle.

Among the four non-cyclic bleeding patterns there are two that are desirable bleeding patterns and two that are undesirable. Bleeding pattern number 3 in Figure 5 shows amenorrhea, a pattern typical of post menopausal women taking continuously combined steroid hormone preparations. Bleeding pattern number 4 in Figure 5 can be interpreted as the pattern of fertile women which start continuous steroid hormonal treatment, e.g. for the treatment of endometriosis. The natural cyclic bleeding ceases during the first month of treatment and thereafter reaches amenorrhea as in pattern number 3. The bleeding patterns 5 and 6 in Figure 5 both show a high frequency of undesirable spotting or bleeding. Pattern 6 is worse than pattern 5.

## Discussion

Several suggestions have been made in the literature over the past years for the analysis of bleeding diaries [16-18], all of them were based on theoretical considerations. To our knowledge, this is the first analysis to recognise patterns in bleeding diaries using empirical methods.

A hierarchical agglomerative cluster analysis with the method of Ward yielded six bleeding patterns which allow for a straightforward clinical interpretation, either desirable or undesirable bleeding pattern for a certain treatment target. In contrast the single, average and complete linkage methods chained the data into a single cluster, which was not interpretable from a gynaecologic point of view.

## Conclusion

Using this cluster analysis with the method of Ward, treatments (medications and devices) having an impact on bleeding can be easily compared and categorized. This analysis is independent of the treatment's route of administration (oral, transdermal, vaginal, intrauterine) and the duration of treatment. Hormonal and non-hormonal treatments can be easily compared. However, this method is only useful in large clinical trials to characterize a new product's bleeding pattern but it is not meaningful for the physician treating an individual patient.

Since currently various innovative long-cycle contraceptive regimen are in development, this method might be useful to compare bleeding pattern from different studies using one uniform method.

## Competing interests

CG and JW are fulltime employees of Bayer Schering Pharma AG, Berlin, Germany and JE is a fulltime employee of Bayer Inc., Toronto, Ontario, Canada. Bayer Schering Pharma AG funded this study and the article processing charges.

GK declares that he has no competing interests.

## Authors' contributions

JE, CG, and JW designed the study. CG performed the statistical analyses. CG and JE drafted the manuscript. GK and JW critically reviewed the draft manuscript. All authors read and approved the final manuscript.

## Pre-publication history

The pre-publication history for this paper can be accessed here:


